# A standardized nomenclature for resistance-modifying agents in the Comprehensive Antibiotic Resistance Database

**DOI:** 10.1128/spectrum.02744-23

**Published:** 2023-11-16

**Authors:** Keaton W. Smith, Brian P. Alcock, Shawn French, Maya A. Farha, Amogelang R. Raphenya, Eric D. Brown, Andrew G. McArthur

**Affiliations:** 1 David Braley Centre for Antibiotic Discovery, McMaster University, Hamilton, Ontario, Canada; 2 Michael G. DeGroote Institute for Infectious Disease Research, McMaster University, Hamilton, Ontario, Canada; 3 Department of Biochemistry and Biomedical Sciences, McMaster University, Hamilton, Ontario, Canada; University at Albany, Albany, New York, USA

**Keywords:** antibiotic adjuvants, resistance-modifying agents, nomenclature, standardization, mechanisms of action

## Abstract

Resistance-modifying agents have been historically underrepresented in the Comprehensive Antibiotic Resistance Database (CARD). In the process of curating over 60 new molecules into CARD’s Antibiotic Resistance Ontology, the limitations of current classification and nomenclature for these molecules were addressed. We propose a new standardized nomenclature based on mechanism of action, including inhibitors of antibiotic resistance mechanisms, adjuvants enhancing antibiotic entry, adjuvants inhibiting antibiotic removal, adjuvants that alter cell physiology, and host-related antibiotic adjuvants.

**IMPORTANCE:**

While increasing rates of antimicrobial resistance undermine our current arsenal of antibiotics, resistance-modifying agents (RMAs) hold promise to extend the lifetime of these important molecules. We here provide a standardized nomenclature for RMAs within the Comprehensive Antibiotic Resistance Database in aid of RMA discovery, data curation, and genome mining.

## OBSERVATION

The Comprehensive Antibiotic Resistance Database (CARD) is an expertly curated ontologically structured database of antibiotic resistance determinants, mechanisms, and associated molecules (1). Since its inception, CARD’s primary effort has been to maintain an up-to-date collection of reference molecular sequences, antibiotics, and bioinformatics models associated with bacterial antimicrobial resistance. Other than well-known β-lactamase inhibitors (BLIs), until recently, resistance-modifying agents (RMAs) were largely overlooked by CARD’s curators. These agents, also commonly called adjuvants, are molecules that do not exhibit direct or sufficiently efficacious antibiotic activity themselves but rather increase or rescue susceptibility to *bona fide* antimicrobials in otherwise resistant pathogens (2, 3). Herein, we report recent updates to CARD’s Antibiotic Resistance Ontology (ARO) to better classify adjuvants as a major curation effort resulting in the addition of over 60 new molecules to the ARO. In doing so, we propose a new standardized nomenclature for these important molecules.

### Categorization of RMAs

**Fig 1:**
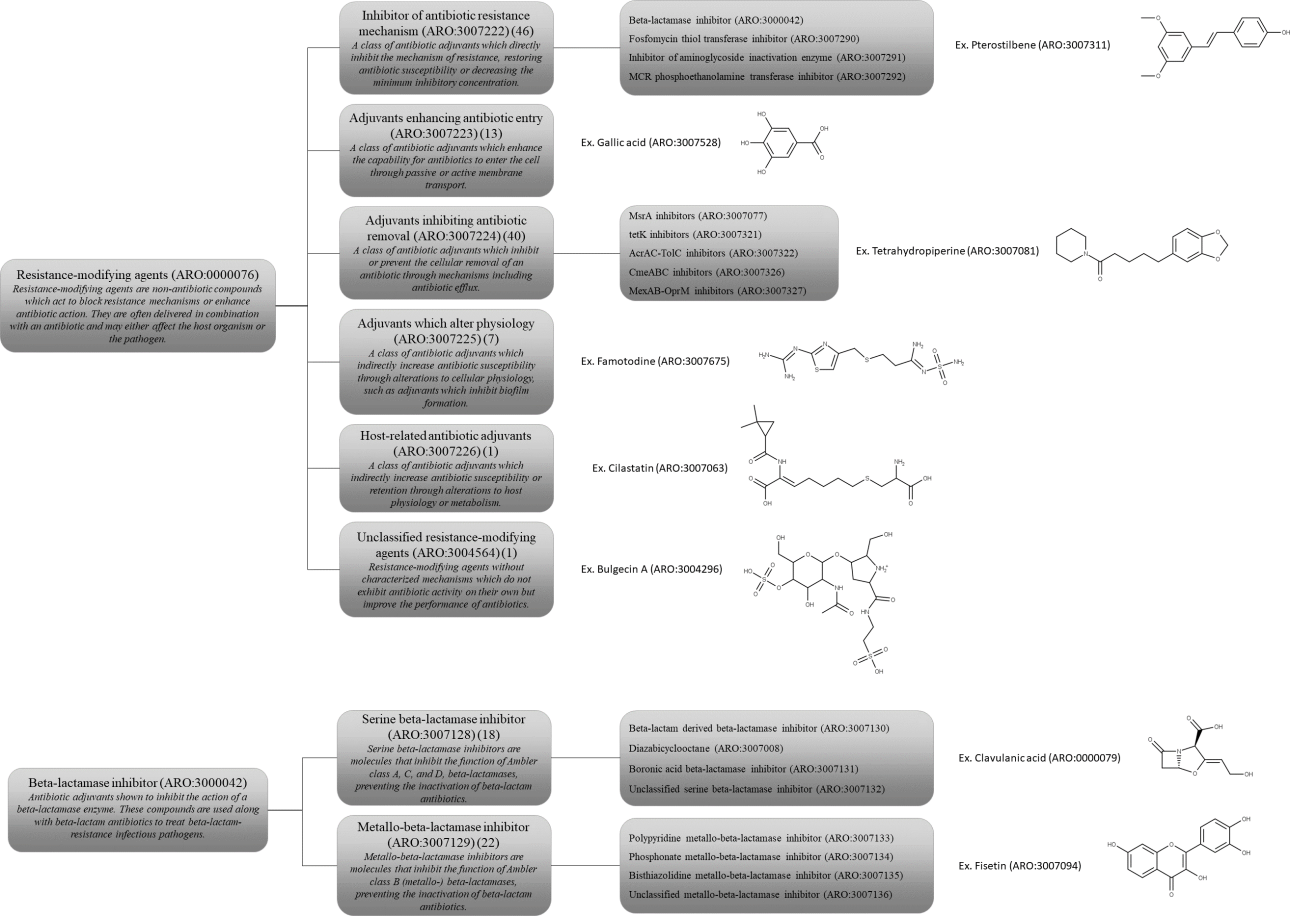
(Top) Breakdown of the high-level categories of RMAs in the ARO. The definition of each category is paraphrased in italics, and the total number of ARO terms belonging to each group is indicated in parentheses, with major subcategories listed where applicable. Terms on the left are categorically higher than those to which they are connected. The names and structures of example molecules are shown to the right of each major category. (Bottom) β-Lactamase inhibitors are further divided on whether they target serine or metallo-β-lactamases. These groups are subcategorized based on the chemical structure.

RMAs represent one of the seven major branches of the ARO and have been broadly divided into six main categories based on their mechanism of action (Fig. 1). These categories consist of (i) inhibitors of antibiotic resistance mechanisms, which include molecules such as BLIs that directly or indirectly inhibit antibiotic inactivation enzymes; (ii) adjuvants enhancing antibiotic entry, which include molecules that increase bacterial membrane permeability and allow antibiotics to more readily enter the cell; (iii) adjuvants inhibiting antibiotic removal, which include molecules such as efflux pump inhibitors that decrease the rate of antibiotic removal from the cell; (iv) adjuvants that alter cell physiology, which include molecules that disrupt antibiotic tolerance mechanisms such as bacterial biofilm formation and quorum sensing; (v) host-related antibiotic adjuvants, which include molecules that interact with host physiology or metabolism to improve the bioavailability or prolong the half-life of antibiotics; and (vi) unclassified RMAs, which include adjuvants with unique or unknown modes of action (4). Some of these categories are further divided into specific functional groupings (Fig. 1). For example, inhibitors of antibiotic resistance mechanisms are separated into subcategories based on the antibiotic-inactivating protein targeted by the adjuvant. BLIs in particular have undergone extensive subcategorization since they constitute the bulk of the clinically available and experimental adjuvants curated in CARD (Fig. 1). These molecules are broadly separated based on their inhibition profiles, which are typically exclusive to either serine β-lactamases (Ambler classes A, C, and D) or zinc metallo-β-lactamases (Ambler class B) (5). Serine BLIs are further divided into β-lactam-derived, diazabicyclooctane, boronic acid, and unclassified serine BLI subcategories, and metallo-BLIs are further divided into polypyridine, phosphonate, bisthiazolidine, and unclassified metallo-β-lactamase inhibitor subcategories. The ARO framework allows BLIs with broad spectra, such as taniborbactam, to exist within subcategories of both serine and metallo-β-lactamase inhibitor groupings (6). Overall, the ARO framework does not limit adjuvants to any single category, which more accurately reflects the multifunctional nature of these molecules. Several bacterial biofilm disruptors, for instance, fit into several ARO categories since they act through the inhibition of antibiotic efflux pumps (7).

### Curation of RMAs

**Fig 2:**
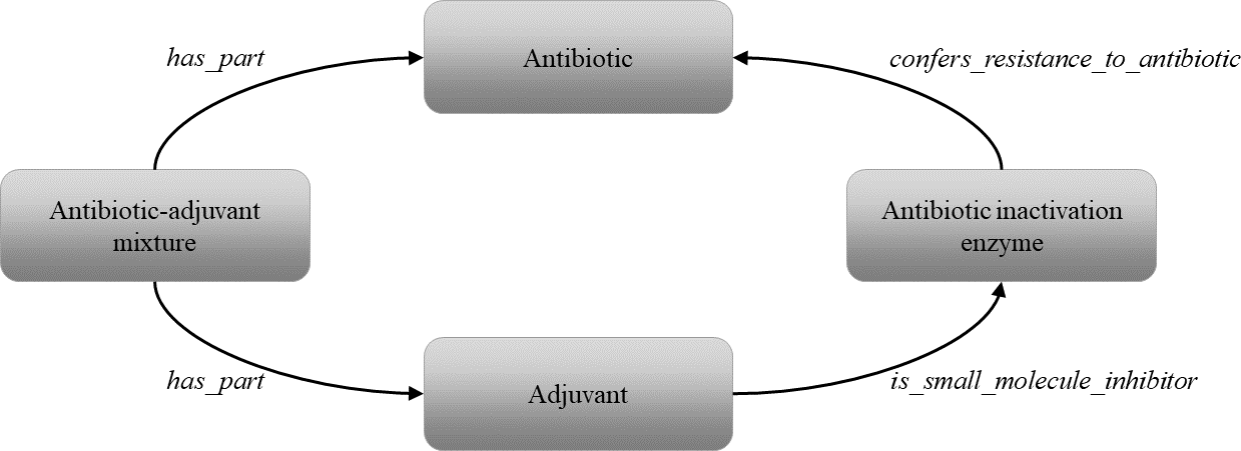
The *is_small_molecule_inhibitor* relationship offers an ontological linkage between adjuvants, antibiotics, and a theoretical antibiotic inactivation enzyme, giving these agents a functional description within the context of the ARO.

Terms in the ARO form a dynamic, interconnected knowledge network via the use of ontological relationships, and these relationships support machine readability and describe the classifications and processes that exist between entries (8). Currently in the ARO, *confers_resistance_to_antibiotic* is a primary relationship between resistance determinants and the antibiotics to which they provide resistance, e.g., salC *confers_resistance_to_antibiotic* clindamycin (9). Furthermore, antibiotic-adjuvant combinations present in CARD possess *has_part* relationships with each individual component of the mixture, e.g., ticarcillin-clavulanic acid *has_part* ticarcillin and *has_part* clavulanic acid. Until the overhaul of the RMA branch, a gap had existed between adjuvants and the rest of the ARO, leading to the addition of the *is_small_molecule_inhibitor* functional relationship (Table 1), which connects inhibitors of antibiotic resistance mechanisms to their specific determinant targets (Fig. 2), e.g., avibactam *is_small_molecule_inhibitor* of TEM-1 and several more β-lactamases. The addition of this relationship term has elevated RMAs to the same level of ontological connectivity within the ARO as antibiotics.

**TABLE 1:** Examples of RMAs sampled from the variety of subcategories in CARD, including their ARO accession, classification, and targeted resistance determinants

Inhibitor of antibiotic resistance mechanism	ARO accession	Type	Class	is_small_molecule_inhibitor
Tazobactam	ARO:0000077	β-lactam-derived β-lactamase inhibitor	serine β-lactamase inhibitor	CMY-54 CTX-M-1 + others OXA-2 + others SHV-1 + others TEM-1 + others
Avibactam	ARO:3000588	diazabicyclooctane	serine β-lactamase inhibitor	ACT-1 CMY-8 + others CTX-M-15 + others IMP-4 KPC-2 + others MOX-1 OXA-1 SHV-1 + others TEM-1 + others
Vaborbactam	ARO:3004380	boronic acid β-lactamase inhibitor	serine β-lactamase inhibitor	CMY-2 CTX-M-3 + others DHA-1 KPC-2 + others MIR-1 NmcA SHV-5 + others SME-2
Bispicen	ARO:3005201	polypyridine metallo-β-lactamase inhibitor	metallo-β-lactamase inhibitor	NDM-1 VIM-1
Risedronate	ARO:3007095	phosphonate metallo-β-lactamase inhibitor	metallo-β-lactamase inhibitor	NDM-1
L-CS319	ARO:3007367	bisthiazolidine metallo-β-lactamase inhibitor	metallo-β-lactamase inhibitor	NDM-1
Emerione A	ARO:3007076	unclassified metallo-β-lactamase inhibitor	metallo-β-lactamase inhibitor	NDM-1
Phosphonoformate	ARO:3007306	-	fosfomycin thiol transferase inhibitor	FosA

Updates to the ARO for RMAs prompted an extensive literature review, a concerted effort to standardize higher-order nomenclature for these molecules, and a curation effort to populate these newly created groupings within the ARO. This led to 64 molecules ultimately identified for curation, including many multifunctional adjuvants. Most notably, experimental and preclinical BLIs such as nacubactam, zidebactam, and ledaborbactam were added to CARD along with several repurposed medications capable of increasing bacterial membrane permeability, including metformin and pentamidine (10 – 12). Plant-derived natural products such as flavonoids were particularly represented both as bacterial efflux pump inhibitors and as disruptors of biofilm formation and quorum sensing (13). The total number of adjuvant molecules present in CARD increased to 103 overall.

### Conclusion

Given the existing rates of antimicrobial resistance, RMAs offer considerable hope for delaying loss of clinical and veterinary use of a broad range of antimicrobials and for revitalizing research into drugs that could pass clinical trials by lowering breakpoint concentrations to avoid adverse toxicology. Yet, to date, there has been no definitive classification of RMAs. The most appreciated classification has been the distinction between Class I (act on pathogen) and Class II (act on host) adjuvants (14). However, it is important to recognize that there are many different ways for a molecule to be an RMA, and this binary classification is too broad for clear description of these molecules and ignores the biology underlying a diverse range of mechanisms of action. CARD’s proposed six categories of RMA are intended to be all-inclusive of potential mechanisms of action of RMAs, and we encourage the community to adopt these classifications as new standards for describing new discoveries in future work. We anticipate that with continued innovation and discovery of RMAs, the number of described mechanisms and categories in the ARO will increase. As antibiotics have their general classes and targets, it is important for RMAs to follow suit in aid of discovery, data curation, and genome mining.
